# FXYD domain containing ion transport regulator 5 (FXYD5) silencing promotes cell viability and alleviates inflammatory response in cerulein-induced AR42J cells by blocking JAK2/STAT3 signaling pathway

**DOI:** 10.1080/21655979.2021.2023795

**Published:** 2022-01-18

**Authors:** Licheng Ding, Jie Li

**Affiliations:** a Department of Emergency, Affiliated Hospital of Jiangnan University, Wuxi, Jiangsu P.R. China; b Department of Emergency, Maternal and Child Health Hospital of Hubei Province, Tongji Medical College, Huazhong University of Science and Technology, Hubei Wuhan, P.R. China

**Keywords:** FXYD5, JAK2/STAT3, acute pancreatitis, inflammation

## Abstract

Acute pancreatitis (AP), which causes severe morbidity and mortality, is a heavy burden for people clinically and financially. This study was designed to explore the mechanism of AP and try to find effective therapies against AP. The expression of FXYD5 was interfered by performing transfection. RT-qPCR and Western blot were utilized to measure FXYD5 expression. In addition, the viability, apoptosis and inflammatory response were evaluated using CCK-8, TUNEL and ELISA, respectively. Moreover, Western blot was employed to measure the expressions of apoptosis-, inflammation- and signaling pathway-related proteins. FXYD5 was found to be overexpressed in AP patients and AP cell model. The results showed that in cerulein-induced AR42J cells, cell viability was remarkably increased, and apoptosis was inhibited compared to the normal FXYD5-expressing group because FXYD5 was downregulated. Similarly, in such cells, interference with FXYD5 significantly suppressed the inflammatory response. In addition, Western blot analysis revealed that JAK2/STAT3 signaling was also strongly inhibited by FXYD5 interference. However, the effect of FXYD5 downregulation was reversed upon simultaneous activation of JAK2/STAT3 signaling. In conclusion, downregulation of FXYD5 could promote cell viability and alleviate inflammatory response in cerulein-induced AP via blocking JAK2/STAT3 signaling pathway.

## Introduction

As an inflammatory disease of the pancreas, acute pancreatitis contributes to high morbidity and mortality because of the advancement of pancreatic and extra-pancreatic necrosis, subsequent infection and multisystem organ failure (MOF) [1–3]. It is noted that 80% of patients with acute pancreatitis can self-resolve without serious complications, but up to 20% of patients will have complications [4]. With mild symptoms, acute pancreatitis has an incidence varying from 5 to 80 cases per 100,000 residents every year, accounting for 10–15% of overall mortality rate [5]. Up to now, the etiology and pathogenesis of acute pancreatitis remain unclear, and thus it is of great urgency to determine the multi-causality of this disease and find effective methods for the treatment of acute pancreatitis.

FXYD5, also known as dysadherin or RIC, is a type 1 transmembrane protein and a member of FXYD family with unique structure and function [6]. Many studies have testified that FXYD5 was upregulated in metastatic tumors, which suggested that FXYD5 acted as an oncogenic marker [7–10]. Besides, FXYD5 could regulate inflammation. For example, FXYD5 increased the inflammatory response in LPS-induced epithelial cells via upregulating its expression [11]. It was also found that FXYD5 expression was elevated in the lungs of patients suffering from acute lung injury [12]. Although FXYD5 has been investigated in many diseases, its role in acute pancreatitis is unknown, especially its effects on the inflammatory response of acute pancreatitis. Therefore, we conducted this study.

As a member of protein-tyrosine kinases, Janus kinase 2 (JAK2) participates in the proliferation, differentiation and viability of cells [13]. Signal transducer and activator of transcription 3 (STAT3) belongs to the STAT family, the phosphorylation of which could be induced via activating JAK2 [14]. An increasing number of evidence has shown that JAK2/STAT3 pathway elicits the inflammatory response in numerous diseases and its activation is involved in oncogenic processes, such as proliferation, viability and angiogenesis of some diseases [15,16]. According to GEPIA database (http://gepia.cancer-pku.cn/), FXYD5 is positively correlated with JAK2 and STAT3 in the pancreas. In view of this, we wondered whether FXYD5 downregulation could inhibit the inflammatory response in acute pancreatitis via regulating JAK2/STAT3 pathway.

## Materials and methods

### Clinical samples

The study was approved by the Medical Ethics Committee of Maternal and Child Hospital of Hubei Province and informed consent was obtained from patients for all samples. The serum samples from 20 AP patients and 20 healthy people were collected, respectively, and the expression levels of FXYD5 in serum were analyzed by reverse transcription-quantitative PCR analysis.

### Cell culture and treatment

Rat pancreatic acinar AR42J cells sourced from American Type Culture Collection were incubated in Ham’s F-12 K medium (Gibco, Thermo Fisher Scientific Inc.) with 12% fetal bovine serum (FBS) at 37°C in a humidified atmosphere with 5% CO_2_. To construct the AP cell model, AR42J cells were treated with 10 nM cerulein for 24 h at 37°C with 5% CO_2_. To explore the underlying mechanism of JAK2/STAT3 signaling pathway, colivelin (CLN), an agonist of JAK2/STAT3, was utilized to treat AR42J cells.

### Cell transfection

To knock down the expression of FXYD5 in AR42J cells, small interfering RNA (si-RNA) targeting FXYD5 (si-FXYD5-1 and si-FXYD5-2) and corresponding negative control (si-NC) were made and compounded by Genscript Biotech Corp. si-FXYD5-1, si-FXYD5-2 and si-NC were respectively transduced into AR42J cells (2x10^5^ cells/well in 6-well plates) using Lipofectamine® 2000 transfection reagent (Invitrogen; Thermo Fisher Scientific, Inc.), according to the manufacturer’s instruction. Transfection efficiency was detected via RT-qPCR at 48 h after transfection.

### Reverse transcription-quantitative PCR (RT-qPCR) analysis

Total RNA from AR42J cells was isolated with Trlzol® reagent (Thermo Fisher Scientific, Inc.). Then, the extracted RNA was synthesized into complementary DNA (cDNA) with the application of a cDNA Synthesis Kit (Invitrogen, Thermo Fisher Scientific, Inc.). Subsequently, qPCR reaction on ABI 7500 quantitative PCR instrument was performed using SYBR Premix Ex Taq reagents (Takara). The relative gene expression was determined with 2^−ΔΔCt^ method. The following primers pairs: FXYD5 forward 5ʹ-GTGTCTCCTCACTATTGTCGC-3ʹ and reverse 5ʹ-GGTCCGCTGTAAAAATGGATGT-3ʹ; GAPDH forward 5ʹ- TGGCCTTCCGTGTTCCTAC-3ʹ and reverse 5ʹ-GAGTTGCTGTTGAAGTCGCA-3ʹ.

### Western blotting

Proteins from AR42J cells were extracted using radioimmunoprecipitation assay lysing solution and quantified using the Bicinchoninic acid (BCA) methods. Separated from 12% gel with sodium dodecyl sulfate polyacrylamide gel electrophoresis (SDS-PAGE), the proteins were transferred to a polyvinylidene fluoride (PVDF) membrane. After inhibition with 5% skim milk, the membranes were incubated with primary antibodies at 4°C overnight. On the next day, the secondary antibody was applied to incubate the membranes for 1 h. Finally, the protein bands were imaged by enhanced chemiluminescence (Invitrogen; Thermo Fisher Scientific, Inc.). The following primary antibodies were used: anti-FXYD5 (1:500; Proteintech^TM^), anti-Bcl-2 (1:1000; Abcam), anti-Bax (1:1000; Abcam), anti-Cox2 (1:1000; Abcam), anti-iNOS (1:1000; Abcam), anti-p-JAK2 (1:1000; Abcam), anti-JAK2 (1:2000; Abcam), anti-p-STAT3 (1:1000; Abcam), anti-STAT3 (1:1000; Abcam) and anti-GAPDH (1:500; Abcam).

### Cell counting Kit-8 (CCK-8) assay

AR42J cells were inoculated into 96-well plates and treated with cerulein for 24 h. Subsequently, 10 μl of CCK-8 reagent was added into each well and the cells were fostered for another 4 h. Finally, the absorbance at 450 nm was determined with a microplate reader (BioTek).

### TUNEL assay

The effects of FXYD5 silence on apoptosis of AR42J cells were assessed using a TUNEL assay kit (Invitrogen; Thermo Fisher Scientific Inc.). In brief, AR42J cells were fixed with 4% paraformaldehyde, permeabilized with 0.25% Triton‐X 100 and labeled with TUNEL for 60 min at room temperature. Thereafter, DAPI was employed to stain cell nuclei for 10 min. Finally, the image of apoptotic cells was photographed by fluorescence microscopy (Nikon).

### ELISA assay

With the aim of detecting the release of inflammatory cytokines, the ELISA Kits (Beyotime, Haimen, China) were employed to evaluate the levels of (tumor necrosis factor-α), IL-1β (Interleukin-1β) and IL-6 (Interleukin-6), respectively. The optical density at 450 nm was recorded with a microplate reader (Bio-Rad, USA), and TNF-α, IL-1β and IL-6 levels were calculated using the standard curve.

### Statistical analysis

All data collected from the experiments were analyzed with GraphPad Prism 8.0 software (GraphPad Software, Inc.) and displayed as mean ± standard deviation (SD). Following a one-way ANOVA analysis, Tukey’s post hoc test was carried out to conduct statistical comparisons. Unpaired Student’s test was used to count the differences between the two groups of data. P value <0.05 was viewed to be of statistical difference.

## Results

### FXYD5 was upregulated in AP patients and AP cell model

**Figure 1.**
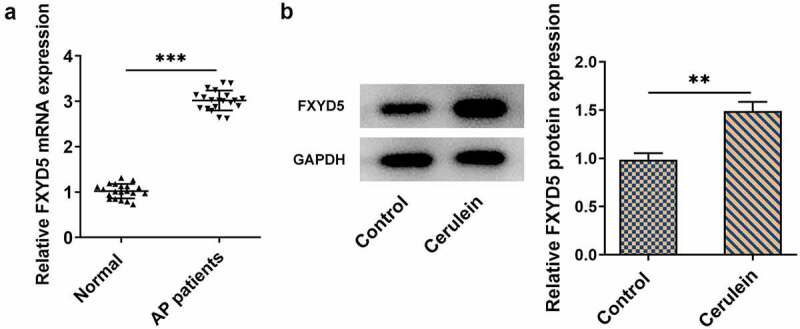
FXYD5 was upregulated in AP patients and AP cell model. (a) The mRNA expression of FXYD5 was detected using RT-qPCR. (b) The protein expression of FXYD5 was detected using Western blot. **P < 0.01 and ***P < 0.001.

To figure out the role of FXYD5 in acute pancreatitis, the mRNA and protein expression of FXYD5 were firstly detected using RT-qPCR and Western blot, respectively. As Figure 1(a) demonstrated, the mRNA expression of FXYD5 was greatly upregulated in AP patients compared with normal group. Moreover, FXYD5 protein expression gained a huge growth in cerulein-induced AR42J cells (Figure 1(b)). The above results revealed that FXYD5 was upregulated in AP patients and AP cell model.

### FXYD5 silence promoted the growth of cerulein-induced AR42J cells

**Figure 2.**
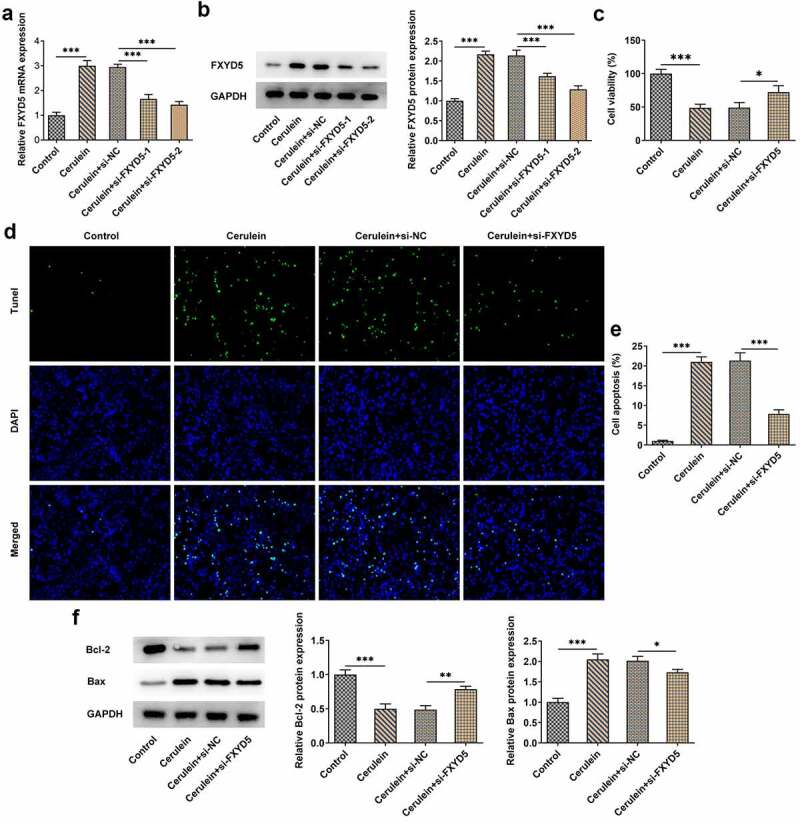
FXYD5 silence promoted the growth of cerulein-induced AR42J cells. (a) The mRNA expression of FXYD5 was measured using RT-qPCR. (b) The protein expression of FXYD5 was measured using Western blot. (c) The cell viability was detected using CCK-8. (d) The apoptosis was detected using TUNEL. (e) Quantitative statistical results of cell apoptosis. (f) The expressions of apoptosis-related proteins were detected using Western blot. *P < 0.05, **P < 0.01 and ***P < 0.001.

With the aim of silencing FXYD5 expression in AR42J cells, si-RNA targeting FXYD5 (si-FXYD5-1 and si-FXYD5-2) was engineered and transfected into AR42J cells. Subsequently, RT-qPCR and Western blot were used to measure the expression of FXYD5. Results from Figure 2(a-b) implied that induction of cerulein resulted in a significant increase for FXYD5 mRNA and protein expression compared to control group. However, the increased FXYD5 expression in cerulein-induced AR42J cells was downregulated after transfection with si-FXYD5. It was noted that FXYD5 showed the lowest expression in Cerulein+si-FXYD5-2 group. Therefore, si-FXYD5-2 was adopted for subsequent experiments (written as si-FXYD5). As Figure 2(c) demonstrated, the decreased cell viability caused by cerulein induction was increased by FXYD5 silence. Cerulein induction increased the apoptosis of AR42J cells, while FXYD5 silence reversed the promotive effects of cerulein, as evidenced by the decreased apoptosis in cerulein+si-FXYD5 group (Figure 2(d-e)). Moreover, compared with control group, cerulein induction downregulated Bcl-2 expression but upregulated Bax expression, which was then reversed by FXYD5 silence (Figure 2(f)).

### FXYD5 silence inhibited the inflammatory response of cerulein-induced AR42J cells

**Figure 3.**
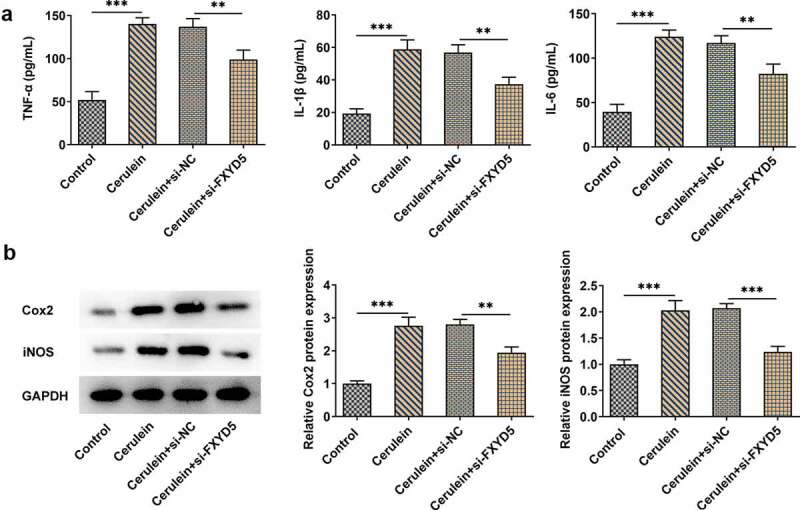
FXYD5 silence inhibited the inflammatory response of cerulein-induced AR42J cells. (a) The inflammatory response was detected using ELISA. (b) The expressions of inflammation-related proteins were measured using Western blot. **P < 0.01 and ***P < 0.001.

With the adoption of ELISA kits, the expressions of inflammatory cytokines, including TNF-α, IL-1β and IL-6 were detected. According to Figure 3(a), cerulein induction significantly increased the TNF-α expression compared to control group, while FXYD5 silence inhibited TNF-α in cerulein-induced AR42J cells. Meanwhile, the trends of IL-1β and IL-6 expression were similar with that of TNF-α. Besides, the expressions of apoptosis-related proteins, including cyclooxygenase 2 (Cox2) and inducible nitric oxide synthase (iNOS), were measured (Figure 3(b)). It was noted that cerulein induction upregulated the expressions of Cox2 and iNOS, while FXYD5 knockdown suppressed the increased Cox2 and iNOS, revealing that FXYD5 silence inhibited the inflammatory response of cerulein-induced AR42J cells.

### FXYD5 silence blocked the activation of JAK2/STAT3 signaling pathway

**Figure 4.**
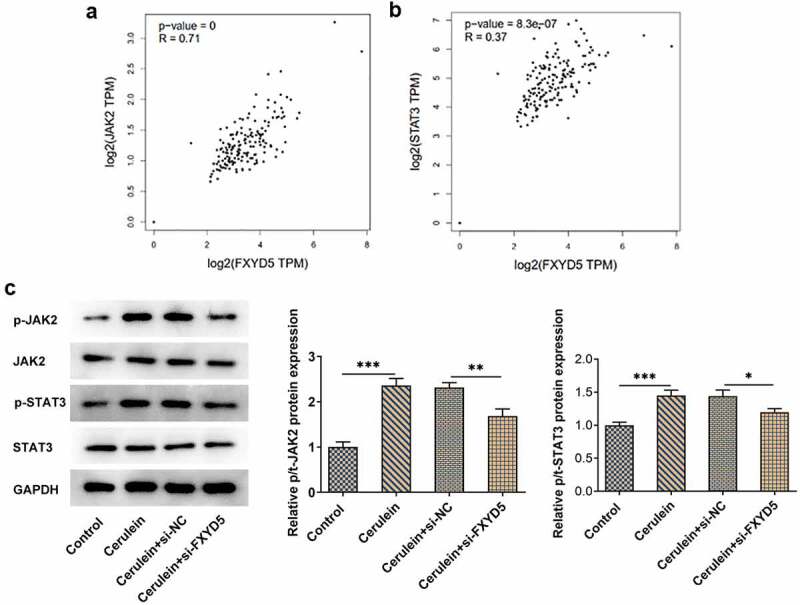
FXYD5 silence blocked the activation of JAK2/STAT3 signaling pathway. The relationship between FXYD5 and JAK2(a)/STAT3(b) signaling pathway. (c) The expressions of JAK2/STAT3 signaling pathway-related proteins were detected using Western blot. *P < 0.05, **P < 0.01 and ***P < 0.001.

According to GEPIA database (http://gepia.cancer-pku.cn/), FXYD5 was positively correlated with JAK2 and STAT3 in the pancreas (Figure 4(a-b)). Moreover, the protein expressions of JAK2/STAT3 signaling were measured by using Western blot. Results demonstrated that cerulein could activate JAK2/STAT3 signaling (Figure 4(c)). Nevertheless, the expressions of p/t-JAK2 and p/t-STAT3 in cerulein-induced AR42J cells were subsequently decreased by FXYD5 silence compared to cerulein+si-NC.

*FXYD5 silence promoted the growth of cerulein-induced AR42J cells via blocking JAK2/STAT3 signaling pathway*

**Figure 5.**
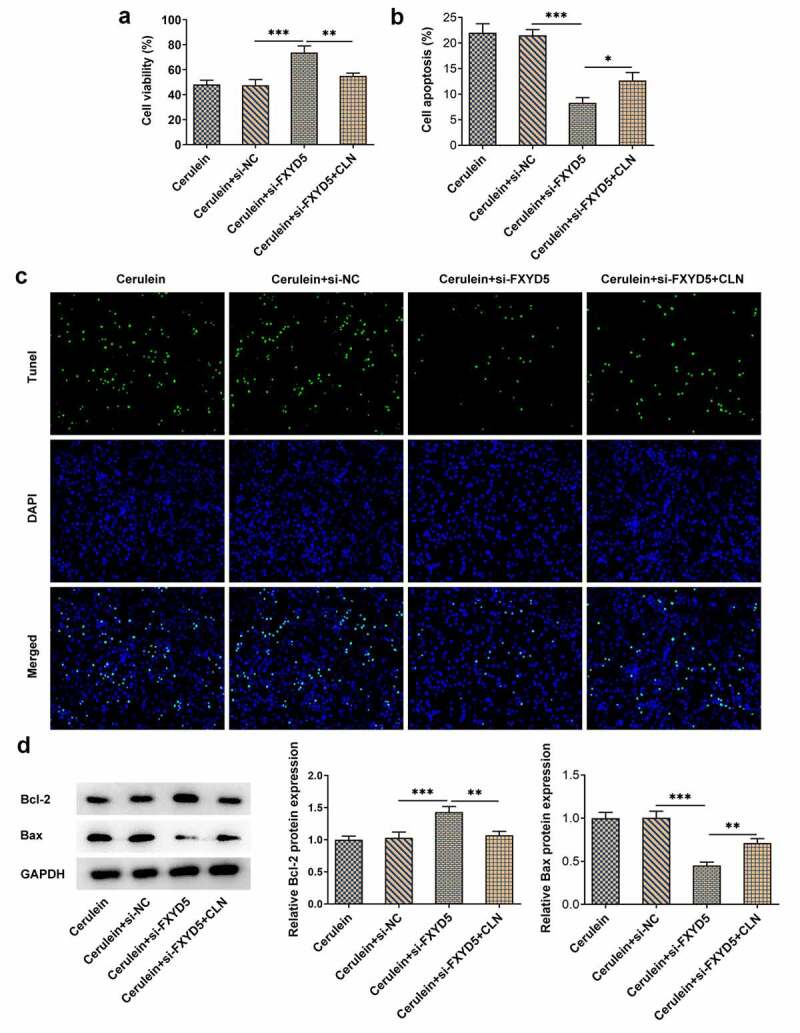
FXYD5 silence promoted the growth of cerulein-induced AR42J cells via blocking JAK2/STAT3 signaling pathway. (a) The cell viability was detected using CCK-8. (b) Quantitative statistical results of cell apoptosis. (c) The apoptosis was detected using TUNEL. (d) The expressions of apoptosis-related proteins were measured using Western blot. *P < 0.05, **P < 0.01 and ***P < 0.001.

In order to explore the mechanism of JAK2/STAT3 signaling pathway, colivelin (CLN), an agonist of JAK2/STAT3 signaling pathway, was employed to treat AR42J cells. Results indicated that FXYD5 silence enhanced the viability of cerulein-induced AR42J cells, while CLN suppressed that increased cell viability (Figure 5(a)). In addition, compared with Cerulein+si-NC, FXYD5 silence inhibited the apoptosis of cerulein-induced AR42J cells, which was then reversed by CLN treatment (Figure 5(b-c)). What is more, FXYD5 silence upregulated Bcl-2 expression but downregulated Bax expression, while CLN exhibited opposite effects on Bcl-2 and Bax, as evidenced by the downregulated Bcl-2 and upregulated Bax expressions in cerulein+si-FXYD5 + CLN group (Figure 5(d)).

*FXYD5 silence inhibited the inflammatory response of cerulein-induced AR42J cells via blocking JAK2/STAT3 signaling pathway*

**Figure 6.**
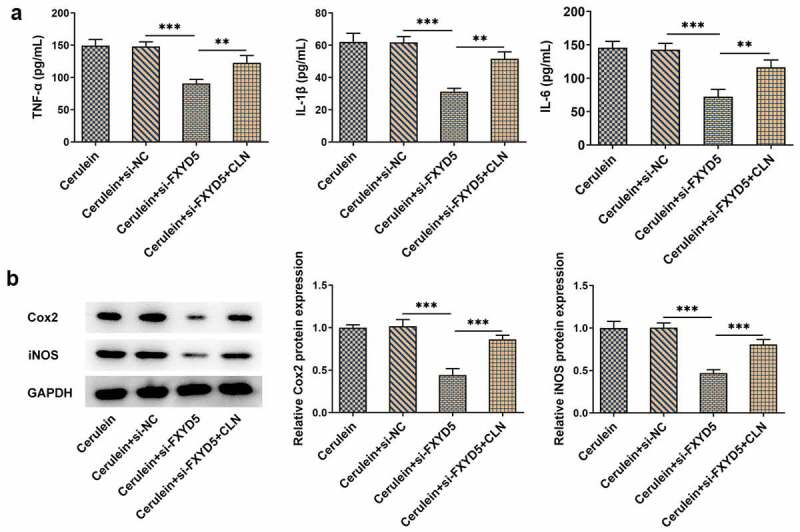
FXYD5 silence inhibited the inflammatory response of cerulein-induced AR42J cells via blocking JAK2/STAT3 signaling pathway. (a) The inflammatory response was evaluated using ELISA. (b) The expressions of inflammation-related proteins were detected using Western blot. **P < 0.01 and ***P < 0.001.

Compared with Cerulein+si-NC, FXYD5 knockdown greatly diminished the expressions of TNF-α, IL-1β and IL-6. However, CLN treatment reversed the inhibitory effects of FXYD5 knockdown on TNF-α, IL-1β and IL-6, as evidenced by the increased TNF-α, IL-1β and IL-6 expressions (Figure 6(a)). As Figure 6(b) showed, the protein expressions of Cox2 and iNOS were increased by CLN treatment in cerulein+si-FXYD5 + CLN group.

## Discussion

As an inflammatory disease of the pancreas, acute pancreatitis is costly every year [17,18]. Despite the fact that the mortality rate of acute pancreatitis in the general population remains unchanged, the incidence rate seems to be increased [19,20]. Thus, it is particularly important to investigate the mechanism of pancreatitis for better treatment. With this in mind, we conducted this study, sparing no efforts to find an effective target for the treatment of acute pancreatitis. In order that the molecular mechanisms can be subsequently better verified in animal models, cerulein-induced rat pancreatic acinar AR42J cells were used as a cellular model for this study. The results showed that FXYD5 was upregulated in acute pancreatitis and FXYD5 silence could promote the growth but suppress the inflammatory response of cerulein-induced AR42J cells. It was also found that FXYD5 silence blocked the activation of JAK2/STAT3 signaling pathway and exhibited suppressive effects on inflammatory response of cerulein-induced AR42J cells via blocking JAK2/STAT3 signaling pathway. Moreover, FXYD5 silence promoted the growth of cerulein-induced AR42J cells via blocking JAK2/STAT3 signaling pathway.

Inflammatory response is a main feature of acute pancreatitis. It has been reported that the production of pro-inflammatory cytokines, including TNF-α, IL-1β and IL-6, is a vital player in the pathogenesis of acute pancreatitis [21,22]. Moreover, the expressions of TNF-α, IL-1β and IL-6 explain why local pancreatic damage evolves to subsequent systemic complications [22]. J Norman et al. held the opinion that IL-1β expression was a marker with 82% accuracy in predicting the severity of acute pancreatitis, which further pointed out the adverse effects of IL-1β in the pathogenesis of acute pancreatitis [23]. Moreover, TNF-α was verified to play a core role in the pathogenesis of inflammatory diseases, particularly in pancreatitis pathogenesis [24]. In the present study, the levels of TNF-α, IL-1β and IL-6 were significantly increased by cerulein induction compared to controls. Meanwhile, inflammation-associated Cox2 and iNOS protein expression was significantly increased after cerulein induction.

FXYD5 is a family of type I plasma membrane proteins [25]. A study reported that FXYD5 silence inhibited the release of inflammatory cytokines in lung injury [26]. Besides, it was noted that FXYD5 was significantly upregulated in pancreatic tissue of mice with acute pancreatitis, which indicated that FXYD5 directly participated in the occurrence of acute pancreatitis [27]. In this study, we found that FXYD5 was greatly increased in AP patients and AP cell model. Furthermore, the silence of FXYD5 promoted the growth of cerulein-induced AR42J cells. However, in the AP cell model, silencing FXYD5 inhibited expression of multiple inflammatory and related factors, including TNF-α, IL-6, IL-1β, Cox2 and iNOS.

The study suggested that JAK2/STAT3 signaling pathway was involved in many inflammatory processes [28]. For example, the inhibition of JAK2/STAT3 pathway could relieve the inflammation in human umbilical vein endothelial cells [14]. What is more, one previous study evidenced that JAK2/STAT3 signaling pathway played a central role in the advancement of acute pancreatitis [29]. In view of this, the role of JAK2/STAT3 signaling pathway in cerulein-induced acute pancreatitis was important to explore. According to GEPIA database, FXYD5 was positively correlated with JAK2/STAT3 signaling pathway, which was consistent with the results in the present study. In addition, activation of JAK2/STAT3 signaling by the inclusion of CLN revealed that the effects produced by silencing FXYD5 were reversed. These results suggest that silencing FXYD5 blocked the activation of JAK2/STAT3 signaling pathway and exhibited desirable effects on growth and inflammatory response in cerulein-induced AR42J cells via blocking JAK2/STAT3 signaling pathway.

## Conclusion

To sum up, FXYD5 knockdown could promote the cell viability and reduce inflammatory response in cerulein-induced pancreatic acinar cells AR42J via inhibiting JAK2/STAT3 signaling pathway. In the future, studies will continue in different cellular and animal models, which hopefully can provide a new idea for the treatment of acute pancreatitis.

## Highlights

1. In cerulein-induced AR42J cells, FXYD5 knockdown promoted cell viability and suppressed inflammatory responses.
2. FXYD5 knockdown inhibits activation of the JAK2/STAT3 signaling pathway.
3. Silencing FXYD5 inhibits the JAK2/STAT3 signaling pathway, thereby suppressing the cerulein-induced inflammatory response in AR42J cells.
